# Transcutaneous Auricular Vagus Nerve Stimulation for Visually Induced Motion Sickness: An eLORETA Study

**DOI:** 10.1007/s10548-024-01088-6

**Published:** 2024-11-02

**Authors:** Emmanuel Molefi, Ian McLoughlin, Ramaswamy Palaniappan

**Affiliations:** 1 School of Computing, University of Kent, Canterbury, UK; 2 ICT Cluster, Singapore Institute of Technology, Singapore, Singapore

**Keywords:** Transcutaneous auricular vagus nerve stimulation, Motion sickness, Electroencephalography, eLORETA, Source localization

## Abstract

Transcutaneous auricular vagus nerve stimulation (taVNS), a non-invasive form of electrical brain stimulation, has shown potent therapeutic potential for a wide spectrum of conditions. How taVNS influences the characterization of motion sickness – a long mysterious syndrome with a polysymptomatic onset – remains unclear. Here, to examine taVNS-induced effects on brain function in response to motion-induced nausea, 64-channel electroencephalography (EEG) recordings from 42 healthy participants were analyzed; collected during nauseogenic visual stimulation concurrent with taVNS administration, in a crossover randomized sham-controlled study. Cortical neuronal generators were estimated from the obtained EEG using exact low-resolution brain electromagnetic tomography (eLORETA). While both sham and taVNS increased insula activation during electrical stimulation, compared to baseline, taVNS additionally augmented middle frontal gyrus neuronal activity. Following taVNS, brain regions including the supramarginal, parahippocampal, and precentral gyri were activated. Contrasting sham, taVNS markedly increased activity in the middle occipital gyrus during stimulation. A repeated-measures ANOVA showed that taVNS reduced motion sickness symptoms. This reduction in symptoms correlated with taVNS-induced neural activation. Our findings provide new insights into taVNS-induced brain changes, during and after nauseogenic stimuli exposure, including accompanying behavioral response. Together, these findings suggest that taVNS has promise as an effective neurostimulation tool for motion sickness management.

**Supplementary Information:**

The online version contains supplementary material available at 10.1007/s10548-024-01088-6.

## Introduction

Motion sickness is a physiological state of agitation that occurs in response to real, perceived, or virtual motion and that can trigger a variety of neural processes (Cohen et al. 2019; Schmäl 2013; Yates et al. 2014). In its most common manifestation, motion sickness is marked by an increased nausea sensation, with the greatest likelihood of vomiting. It has long been posited – and currently widely accepted – that this polysymptomatic condition arises from conflicts in sensory inputs between systems of the brain that govern proprioception, balance (i.e., vestibular function), and vision (Reason and Brand 1975; Reason 1978; Oman 1990, 1991). Of the manifold theories proposed to explain the enigma of motion sickness, what remains consistent is that the vestibular apparatus is noted as a crucial component for malaise to arise.

Because balance is a function of multiple inputs (Angelaki and Cullen 2008), the neurobiology of motion sickness is complex and remains less well understood. However, recent evidence for the existence of “sensory conflict" neurons and their putative role in motion sickness (Oman and Cullen 2014), has provided marked progress in parsing the neurobiological underpinnings of this elusive malady. Furthermore, previous research has pointed to a key role for the nucleus tractus solitarius (NTS), lateral tegmental field (LTF), and parabrachial nucleus (PBN) brainstem areas, in the integration of signals contributing to nausea and vomiting via mapping of neuronal activity during emetic responses (Yates et al. 2014; Lackner 2019); providing greater insight into the neural substrates of motion-induced nausea.

In humans, functional neuroimaging studies have noted a link between brain activation at regions such as the insula (Napadow et al. 2013), precuneus (Kovács et al. 2008), and cuneus (Farmer et al. 2015), with visually induced motion sickness. Other studies concluded that the medial prefrontal cortex (mPFC), and anterior cingulate cortex (ACC), including pregenual anterior cingulate (pgACC) and dorsal anterior cingulate (dACC) cortices, also contribute to atypical brain activity changes in motion-induced nausea (Kim et al. 2011; Napadow et al. 2013; Ruffle et al. 2019). These brain region examples have important implications. In particular, they could potentially be manipulated for therapies.

While protective benefits from mainstay antiemetic compounds based on antihistamines or anticholinergics have been shown effective for motion sickness, these pharmacologic agents often induce undesirable side effects such as a depressed central nervous system, blurred vision, or drowsiness (Lackner 2014). Whether alternative novel therapeutic approaches with the least side effects can be developed to ward off this malady remains an important challenge. With recent advances in neurostimulation methods and technology – for example, transcutaneous auricular vagus nerve stimulation (taVNS), an electrical brain stimulation technique for non-invasive vagal afferent stimulation – new therapeutic avenues for motion-induced malaise may be possible.

taVNS is a transcutaneous auricular alternative to the conventional cervically implanted vagus nerve stimulation (VNS) (George et al. 2000). This electrostimulation modality, together with transcutaneous cervical VNS (tcVNS), operate via the vagus nerve – a paired neural structure consisting of auricular and cervical branches, that can modulate brain function, and activate the parasympathetic (“rest and digest") nervous system. Although taVNS mechanistic underpinnings have largely remained unclear, a common explanation – with examples from brain imaging studies – is that the auricular branch of the vagus nerve provides a pathway for afferent signaling toward the NTS, therefore stimulating outer-ear regions (i.e., cymba concha, tragus, or both, etc.) activates A$\upbeta$-fibers of the vagus, which transmit electrical impulses to brainstem nuclei (Broncel et al. 2020; Butt et al. 2020).

Neuroimaging studies have suggested that taVNS can elicit functional changes in the brain (Badran et al. 2018). For instance, Kraus et al. (2007) observed reduced brain activation in limbic brain regions, including the amygdala, hippocampus, parahippocampal gyrus, and the middle and superior temporal gyri as measured by functional magnetic resonance imaging (fMRI). Additionally, those authors also found increased activation in the insula, precentral gyrus and the thalamus (Kraus et al. 2007). Using electroencephalogram (EEG) signals, Dimitrov and Gatev (2015) implicated taVNS with brain activation at the middle and superior temporal gyri, precuneus, cuneus and left inferior parietal lobule; whereby EEG was used as a proxy to reconstruct the source localized at those observed functional structures, via low-resolution brain electromagnetic tomography (LORETA). Interestingly, these aforementioned brain region examples are shared or overlap with those triggered by motion sickness, suggesting a potential therapeutic approach to manage the syndrome.

As with motion sickness, vestibular migraine has been suggested to be characterized by altered visual-vestibular interactions (Bednarczuk et al. 2019). In line with this, recent research has shown that electrical stimulation of the vagus nerve may be an effective treatment for vestibular symptoms associated with migraine (Beh and Friedman 2019). Further, emerging data provide preliminary evidence that vagal stimulation may attenuate motion sickness symptoms (Molefi et al. 2023c). These studies illustrate the potential that artificial vagus nerve stimulation may hold for inducing symptom-alleviation effects toward malaise by motion sickness.

How the brain represents and responds to transcutaneous stimulation of the vagus nerve at the functional structure level during the development of motion-induced malaise is not known. Here, we obtain 64-channel EEG recordings from an experimental platform designed to visually induce motion sickness, alongside administration of taVNS. We apply an EEG brain source localization method – exact LORETA (eLORETA) – to glean details about functional localization of taVNS. Our hypothesis is that differences in brain response will be evident from taVNS compared to sham.

## Methods

### Participants

To perform sample size estimation for detecting the effect of taVNS on brain activation and behavioral measures of motion sickness in response to nauseogenic stimuli, we conducted a power calculation using G*Power software (v3.1.9.6; Universität Düsseldorf, Germany) (Faul et al. 2007). The estimated sample size needed to detect an effect with $\upalpha = 0.05$ and at least $80\%$ power for a within-subjects design with two repeated brain activity and behavioral measurements was suggested at $n = 34$. To this end, we recruited a total of 45 healthy participants following written informed consent, of whom ($n = 42$) were retained (mean age ± S.D. = 23.7 ± 6.7 years, age range = 18-49 years, 31 females) for further analysis after one participant was excluded due to not enough data, and two due to loss of follow-up. In addition to normal or corrected-to-normal vision, participants free of any medical history of stroke, epilepsy or neurological conditions; not on any medication; not using a pacemaker; having no metal implants, were invited to the study. Furthermore, we used the motion sickness susceptibility questionnaire short-form (MSSQ-Short) (Golding 2006) as a pre-participation screening tool; including participants reporting a percentile score > 60 (corresponding to an MSSQ raw score of 14.36) in the study. This MSSQ percentile threshold is chosen as it suggests recruited individuals will develop moderate nausea reasonably quickly and reliably, as reported in previous studies using similar justification (e.g., LaCount et al. 2011; Sclocco et al. 2016; Toschi et al. 2017). The MSSQ-Short – a condensed version of the early MSSQ (Golding 1998) – prompts participants to recall experiences of nausea or vomiting at childhood (below 12 years of age; MSA), and over the last decade (MSB) following various transport or entertainment modalities. All protocols were approved by the University of Kent Central Research Ethics Advisory Group (ref: CREAG015-12-2021), and conformed to the standards set by the Declaration of Helsinki. Participants were compensated for their participation (£30 Amazon gift voucher).

**Fig. 1:**
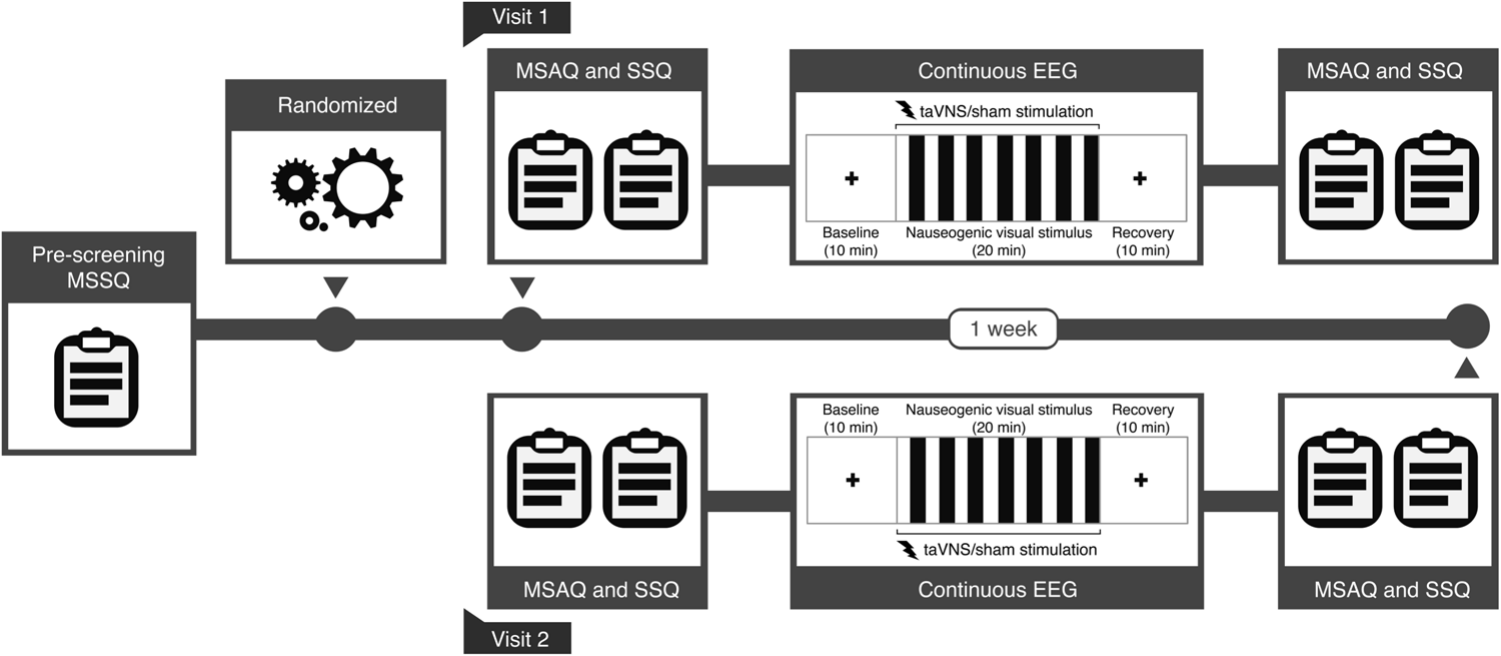
Experimental design and timeline schematics. Participants underwent a pre-screening process which included completion of the motion sickness susceptibility questionnaire (MSSQ). Thereafter, participants were randomized to receive sham or taVNS for their first visit (Visit 1), then receive opposite treatment at 1-week follow-up (Visit 2). On the day of the experiment, participants completed a pre and post motion sickness assessment questionnaire (MSAQ) and simulator sickness questionnaire (SSQ); additionally, participants underwent a baseline period, followed by nauseogenic visual stimulation in synchrony with electrical stimulation, then a recovery period, while electroencephalogram (EEG) signals were recorded

### Experimental protocol

This study employed a randomized, sham-controlled, crossover design (Fig. 1). Participants were required to attend two electrical stimulation sessions, separated by a washout period of at least 1 week, to randomly receive active taVNS or sham control at the initial lab visit, and vice versa at follow-up. During both taVNS and sham sessions, participants were presented with a black crosshair at baseline and recovery for 10 min, respectively. The 10 min of crosshair at baseline denotes 5 min of acclimatization to the experimental room and 5 min of rest state. Between baseline and recovery, participants were exposed to the nauseogenic visual stimulus (see Nauseogenic stimulus) for a maximum of 20 min, coalesced with the administration of electrical stimulation applied at the tragus of the left ear during active taVNS sessions, and earlobe of the left ear during sham control sessions. During this period, participants also provided subjective ratings of nausea, uncued, by pressing a keypad where (0 = “no nausea”), (1 = “mild”), (2 = “moderate”) and (3 = “strong”). To prevent the incidence of vomiting, when on the verge of vomiting, participants could press a button on the keypad to stop the presentation of the nauseogenic visual stimulation; then, the recovery section would be launched automatically. To ensure participant safety, and smooth running of the experiment, the experimenter remained present in the lab but out of view. The three sections, baseline, nauseogenic visual stimulus, and recovery were contiguous (Fig. 1). To obtain brain activity data, we performed continuous EEG from start of baseline through to end of recovery. During the data acquisition period, participants had been informed to minimize body movements and conversation, and to concentrate on the presentation of the stimuli. Finally, to capture symptoms of motion sickness, participants completed a pre- and post-treatment motion sickness assessment questionnaire (MSAQ) (Gianaros et al. 2001), and simulator sickness questionnaire (SSQ) (Kennedy et al. 1993) (see Behavioral measures for details).

### Behavioral measures

We evaluated participant’s experience of motion sickness using the MSAQ, a questionnaire based on 16 symptoms that describe the gastrointestinal, central, peripheral and sopite-related dimensions of malaise. Each individual symptom is rated on a nine-point Likert scale to indicate its severity, 1 being (“not at all") and 9 being (“severely"). To compute the MSAQ total and subscale scores, we followed guidance by Gianaros et al. (2001). The SSQ comprise 16 malaise symptoms which can be categorized into factors indicative of nausea, oculomotor and disorientation. Individual symptoms of the SSQ are recorded on a four-point Likert scale ranging from 0 (“none") to 3 (“severe"). We quantified the SSQ total and subscale scores following recommendations suggested by Kennedy et al. (1993).

### Nauseogenic stimulus

The nauseogenic visual stimulus was custom programmed and run using MATLAB (The MathWorks, Inc., Natick, MA, USA), and the Psychophysics Toolbox Version 3 (Psychtoolbox$-$3.0.19; http://www.psychtoolbox.org) (Brainard 1997; Pelli 1997; Kleiner et al. 2007). A 47-inch LG LCD Widescreen (47LW450U, LG Electronics UK, UK) at a distance filling the participant’s visual field – providing unimpeded field-of-view – was used to present the visual stimulus at a refresh rate of 60 Hz. To induce nausea, the nauseogenic visual stimulus was developed as alternating black and white vertical stripes with left-to-right circular motion at 62.5°/s and presented for a maximum of 20 min or until interruption. Because of the horizontal translation of the visual stripes, participants experience a false perception of translating to the left (i.e., illusory self-motion). This computerised model of nausea induction mimics the visual input provided by the classic rotating optokinetic drum used to provoke motion-induced nausea (Bos and Bles 2004; Levine et al. 2014). Previously, a similar visual stimulus was used for nausea induction (Molefi et al. 2023a, b, c). Neuroimaging studies investigating motion sickness have utilized an fMRI-compatible variant of the stimulus to induce nausea (Napadow et al. 2013; Sclocco et al. 2016).

### Electrical stimulation

To administer taVNS, we delivered electrical current as asymmetric biphasic square-wave pulses with a width of 200 µs at 20 Hz continuously for a maximum duration of 20 min to the tragus of the left ear, and for sham control, to the left earlobe; using the EM6300A TENS device (Med-Fit UK Ltd, Stockport, UK). On average, the electrical stimulation current delivered for taVNS was $5.36 \pm 2.66$ mA (mean ± S.D.), and $5.45 \pm 3.26$ mA for sham control. These stimulation parameters (pulse width and frequency) were chosen matching the protocols of comparable studies (e.g., Beh and Friedman 2019; Tran et al. 2019; Cao et al. 2021). To assess the effectiveness of taVNS experimentally, a control is required for comparison; while a typical control comparison would be the absence of stimulation with otherwise identical settings, the distinct sensation induced by taVNS (reported by all participants) means that a blind test is impossible. Hence the adoption of a sham control (applied at the earlobe), with identical settings and stimulation but at a slightly displaced location separate from the auricular branch of the vagus nerve. A blind test then becomes possible given that participants were not made aware which of the stimulation locations was hypothesised to mitigate nausea. The earlobe is most commonly explored as a sham because it is postulated to be free of vagal innervation (Peuker and Filler 2002; Bermejo et al. 2017; Yakunina et al. 2017). Stimulation current was tested and tailored for each participant prior to the experiment; this was calibrated through a one-up/one-down staircase procedure (Cornsweet 1962), starting with an electric current of 1 mA. All participants reported perception of stimulation without painful sensation. A countdown timer of 20 min was set on the stimulation device, in sync with the maximum duration of exposure to the nauseogenic stimulus; the experimenter turned on the stimulator at nauseogenic visual stimulus onset. Because the electrical stimulation was applied simultaneous to the nauseogenic visual stimulus (Fig. 1), if a participant stopped the nauseogenic visual stimulus due to an impending urge to vomit, the experimenter would immediately switch off the electrical stimulator. None of the participants reported adverse events.

### EEG data acquisition and processing

64-channel EEG data were obtained with a BioSemi ActiveTwo system (BioSemi B. V., Amsterdam, Netherlands) at a sampling rate of 256 Hz. Electrode locations conformed to the extended international 10-20 system. EEG signal processing was performed in MATLAB R2023b using custom scripts, incorporating EEGLAB functions (v2023.0; https://sccn.ucsd.edu/eeglab) (Delorme and Makeig 2004). The raw EEG signals were first notch filtered to remove electrical stimulation-evoked artifact of 20 Hz; and then we performed high-pass (1 Hz) and low-pass (30 Hz) zero-phase response Butterworth IIR filtering; with cutoff frequencies in accordance with EEG processing from previous findings. Channels with noisy activity according to (spectrum = 3 S.D., probability = 3 S.D., and kurtosis = 5 S.D.) threshold measures were removed. To identify and remove eye-blink and muscle artifact components, we performed independent component analysis (ICA) and applied a threshold probability of $80\%$ via ICLabel (v1.3) (Pion-Tonachini et al. 2019). The obtained neuronal sources following ICA were then back-projected to the EEG time series; and spherical interpolation applied. To identify and attenuate major signal outliers and non-brain artifacts, we performed robust principal component analysis (RPCA) (Wright et al. 2009; Candès et al. 2011). To minimise the effect of volume conductivity, surface Laplacian (Perrin et al. 1989) was performed. The artifact-free EEG time series were used to extract 5 min windows at “baseline" (prior to nauseogenic stimulus onset), “stimulation" (prior to nauseogenic stimulus cessation), and “recovery" (following nauseogenic stimulus cessation). Finally, the obtained 5 min windows (“baseline"; “stimulation"; “recovery") were epoched into 8 s segments to obtain smooth power spectral density (PSD) estimate, respectively.

### eLORETA analysis

For EEG brain source localization, we performed the exact low-resolution brain electromagnetic tomography (eLORETA), using the LORETA-KEY software package (v20221229; https://www.uzh.ch/keyinst/loreta) (Pascual-Marqui 2002, 2007; Pascual-Marqui et al. 2011) on the de-noised epoched EEG data from above (see EEG data acquisition and processing). eLORETA – a 3D distributed linear, regularized, weighted minimum-norm inverse solution with exact, zero error localization – is a widely used mathematical tool that estimates neural activity of 6239 voxels (voxel size = 5 mm^3^) of the cortical gray matter using a realistic head model with the MNI152 (Montreal Neurological Institute 152) template (Mazziotta et al. 2001; Fuchs et al. 2002). Previous studies performing other non-invasive brain stimulation techniques – for example, repetitive transcranial magnetic stimulation (rTMS) (Meijs et al. 2024) and transcranial alternating current stimulation (tACS) (Fiene et al. 2020) – have utilized the eLORETA method. Herein, we computed cross-spectra and corresponding frequency domain generators for each participant for five EEG frequency bands: delta (1-4 Hz), theta (4-8 Hz), alpha (8-12 Hz), beta (12-26 Hz), and gamma (26-30 Hz). That is, for each cross-spectrum file, a corresponding .slor file (image of cortical neuronal oscillators) was obtained; statistical analyses were performed on these computed neuronal generators. Note that cross-spectra from the aforementioned EEG frequency bands indicate to us which EEG oscillations (i.e., frequency bands) demonstrate differential neural activity. Consequently, if there are significantly different oscillations, then eLORETA provides an exact localization of neuronal sources responsible for the difference.

### Statistical analysis

To perform statistical analysis for EEG brain source localization, we used the LORETA-KEY software package. The output neuronal generators from above (see eLORETA analysis) were subjected to a Statistical non-Parametric Mapping (SnPM) (Nichols and Holmes 2002) method for correction of multiple comparisons; performing 5000 randomizations with significance threshold of $p < 0.05$, to estimate the empirical probability distribution for the maximum *t*-statistic, under the null hypothesis. Because of the non-parametric nature of this method, its validity does not require Gaussianity assumptions (Nichols and Holmes 2002). We utilized this procedure to test for effects on neural activity using paired *t*-statistic contrasts on log-transformed data, at the sample level. Computed source localization statistical maps were visualized with MRIcroGL (v1.2.20220720; https://www.nitrc.org/projects/mricrogl) (Rorden and Brett 2000). For statistical analyses performed in MATLAB R2023b, voxel intensity values at peak MNI coordinates were extracted using scripts incorporating functions from Statistical Parametric Mapping 12 (SPM12; Wellcome Centre for Human Neuroimaging, London, UK; https://www.fil.ion.ucl.ac.uk/spm). Range normalized MSAQ and SSQ scores were subjected to a two-way repeated-measures ANOVA with the within-subjects factors “time" (pre vs. post) and “stimulation" (taVNS vs. sham) followed by post-hoc paired *t*-tests. Electrical stimulation, and MSSQ data were analysed using non-parametric Wilcoxon signed rank tests. We computed Pearson’s correlation coefficient (expressed as Pearson *r*) for normally distributed data; and Spearman’s rank correlation coefficient (expressed as Spearman $\rho$) for non-normally distributed data. Data are expressed as median and quartiles, or mean ± standard error of the mean (SEM). Normality tests were performed using the Shapiro-Wilk test (see supplementary Table S1). All statistical tests were two-tailed at ($p < 0.05$).

## Results

**Fig. 2:**
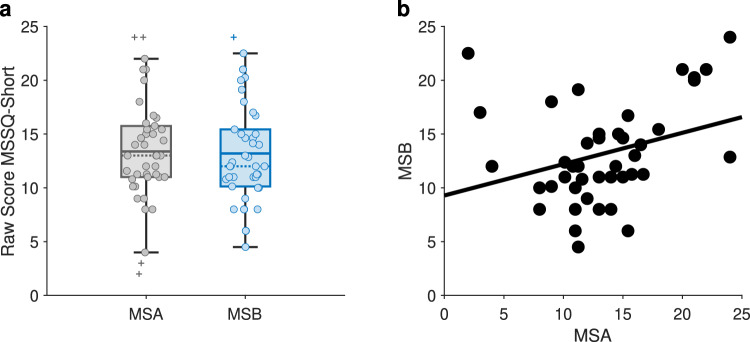
**(a)** Box plot showing MSSQ-Short raw scores of MSA and MSB for all participants. Solid lines indicate mean; dashed lines indicate median. **(b)** Spearman correlation between MSA and MSB where each data point represents a participant (Spearman $\rho$ = 0.35). MSSQ, motion sickness susceptibility questionnaire; MSA, below 12 years of age MSSQ scores; MSB, over the last 10 years MSSQ scores

Participant cohort ($n = 42$) average MSSQ scores were 26.54 (S.D. = 7.82, range = $15.75-48.00$). In order to ascertain greater malaise susceptibility in adolescence for participant cohort herein, we performed a comparison between the MSA and MSB but found no difference ($p = 0.2394$, Wilcoxon signed rank test), with the distributions of the scores presented as box plots with mean and median depicted (Fig. 2a). These MSSQ subscale scores ($\text {MSA}, 13.36 \pm 4.99$; $\text {MSB}, 13.18 \pm 4.68$) were higher than the mean ± S.D. of normative data ($\text {MSA}, 7.75 \pm 5.94$; $\text {MSB}, 5.11 \pm 4.84$) (Golding 2006). Consistent with previous reports, however, there was an association between the MSA and MSB scores (Spearman $\rho$ = 0.35, $p = 0.0251$; Fig. 2b). None of the participants vomited during or after exposure to nauseogenic visual stimulation; however, one participant retched during nausea induction, and was one of the participants absent at a follow-up session. There were no significant differences between electrical current delivered at taVNS and that at sham ($p = 0.8476$, paired-sample *t*-test).

A summary of the MSAQ and SSQ scores is presented in Table 1. There were no differences in the MSAQ subjective responses between sham and taVNS (Table 1). The two-way repeated measures ANOVA revealed a significant effect of stimulation (*F*_1, 41_ $= 32.02,\ p = {1.32}\times 10^{-6}$), time (*F*_1, 41_ $= 63.37,\ p = {7.52}\times 10^{-10}$), and an interaction (*F*_1, 41_ = 4.58, $p = 0.0382$) for the SSQ total scores (see supplementary Fig. S1 for model output and post-hoc analysis). Further, taVNS significantly reduced the SSQ nausea factor scores (two-way repeated measures ANOVA; effect of stimulation, *F*_1, 41_ $= 21.47,\ p = {3.61}\times 10^{-5}$, effect of time, *F*_1, 41_ $= 41.34,\ p = {1.06}\times 10^{-7}$, and stimulation $\times$ time interaction, *F*_1, 41_ = 5.78, $p = 0.0208$; see supplementary Fig. S2 for model output and post-hoc analysis). Notably, no significant effect of order (*sham* $\rightarrow$ *taVNS* vs. *taVNS *$\rightarrow$ *sham*) was observed for SSQ total scores ($p = 0.9510$, unpaired-sample *t*-test), and SSQ nausea factor scores ($p = 0.7426$, unpaired-sample *t*-test).

**Table 1:** Summary of motion sickness assessment questionnaire (MSAQ), and simulator sickness questionnaire (SSQ) total and subscale scores across participants, respectively; in participants receiving transcutaneous auricular vagus nerve stimulation (taVNS) compared with sham. Data are shown as mean ± SEM

	sham		taVNS			
Scores	Pre	Post	Pre	Post	*F*-value	*p*-value
MSAQ						
Total	$0.17 \pm 0.03$	$0.36 \pm 0.04$	$0.14 \pm 0.03$	$0.34 \pm 0.04$	0.09	0.761
Gastrointestinal	$0.06 \pm 0.03$	$0.30 \pm 0.04$	$0.08 \pm 0.03$	$0.29 \pm 0.04$	0.18	0.676
Central	$0.11 \pm 0.04$	$0.35 \pm 0.05$	$0.06 \pm 0.03$	$0.32 \pm 0.04$	0.06	0.814
Peripheral	$0.14 \pm 0.04$	$0.20 \pm 0.04$	$0.09 \pm 0.03$	$0.16 \pm 0.04$	0.10	0.753
Sopite	$0.26 \pm 0.04$	$0.42 \pm 0.04$	$0.18 \pm 0.03$	$0.39 \pm 0.04$	1.05	0.311
SSQ						
Total	$0.24 \pm 0.04$	$0.56 \pm 0.04$	$0.15 \pm 0.03$	$0.37 \pm 0.03$	4.58	$0.038^{*}$
Nausea	$0.21 \pm 0.04$	$0.50 \pm 0.04$	$0.13 \pm 0.03$	$0.29 \pm 0.03$	5.78	$0.021^{*}$
Oculomotor	$0.24 \pm 0.04$	$0.60 \pm 0.03$	$0.19 \pm 0.03$	$0.46 \pm 0.04$	2.79	0.102
Disorientation	$0.15 \pm 0.04$	$0.38 \pm 0.04$	$0.08 \pm 0.03$	$0.37 \pm 0.04$	1.20	0.279

$\hspace{5.0pt}^{*}$ Indicates significant difference in pre and post behavioral changes in response to electrical stimulation ($p < 0.05$) using a two-way repeated-measures ANOVA with the within-subjects factors “time" (pre vs. post) and “stimulation" (taVNS vs. sham). The presented *F* and *p* values are for the interaction effects from a two-way repeated measures ANOVA

**Fig. 3:**
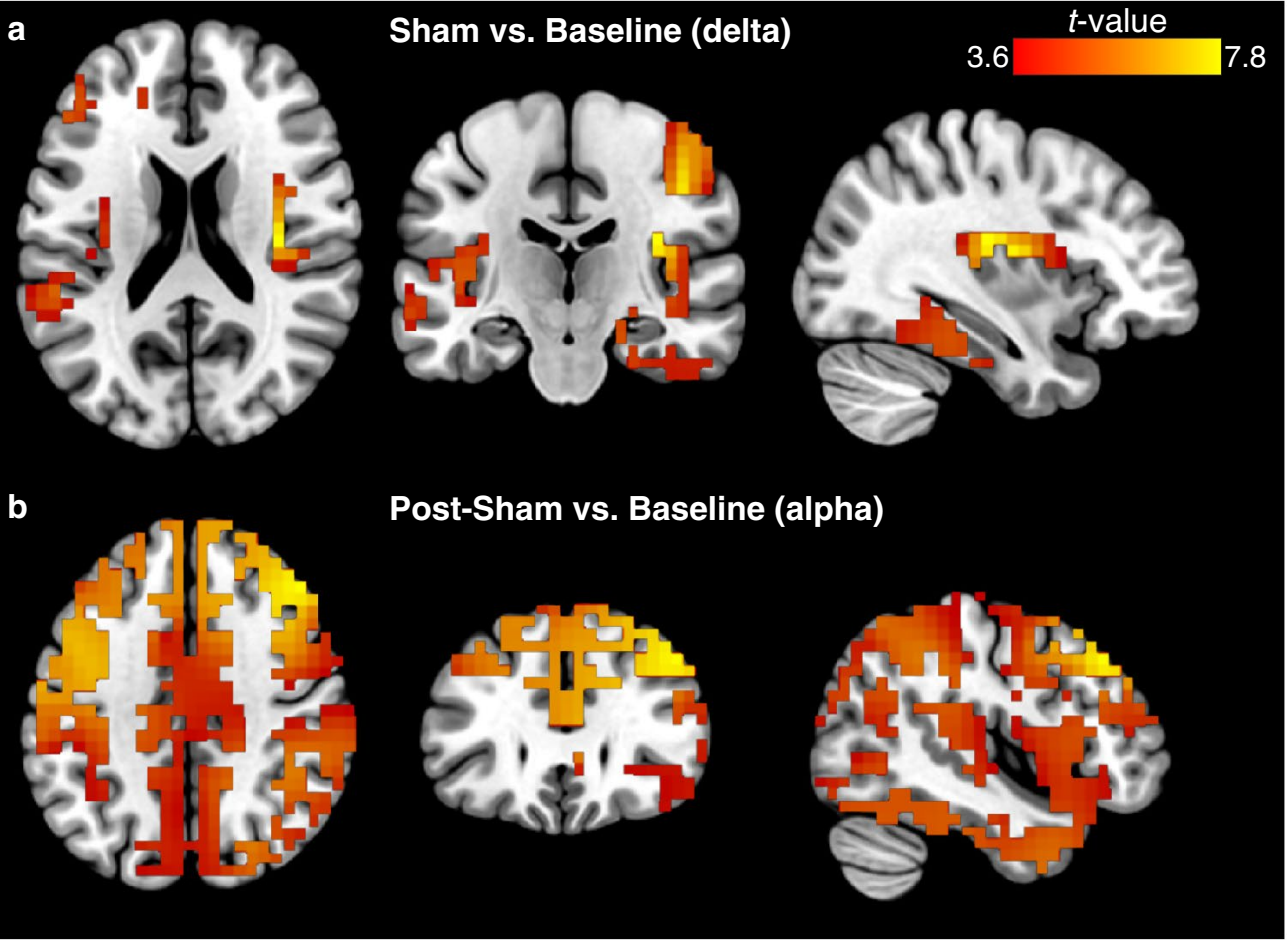
eLORETA of Sham versus Baseline, and Post-Sham versus Baseline contrasts. **(a)** Changes in estimated source activity (delta) between Sham (i.e., during stimulation period) and Baseline were identified in the right insula (BA 13, peak MNI_x,y,z_= 35 -20 20, *t* = 7.83). **(b)** Estimated source activity (alpha) differences between Post-Sham and Baseline were identified at the right middle frontal gyrus (BA 9, peak MNI_x,y,z_= 45 30 40, *t* = 5.70). Slice views of source locations from left to right are axial, coronal, and sagittal images; viewed from top, back, and right. In all of the images, the left side of the brain is shown on the left. BA, Brodmann area

**Fig. 4:**
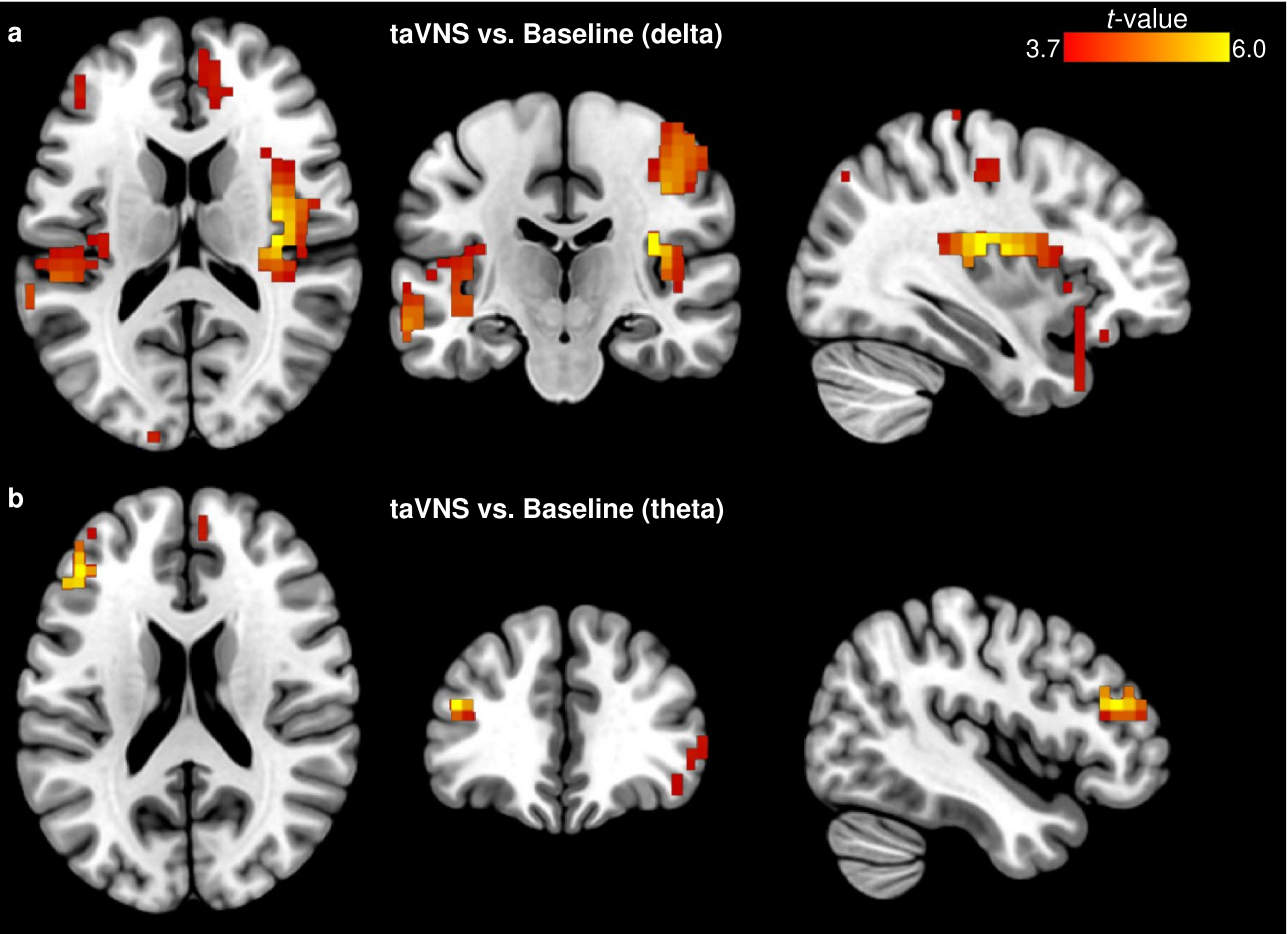
eLORETA of taVNS versus Baseline contrast. **(a)** Differential estimated source activity (delta) at the right insula (BA 13, peak MNI_x,y,z_= 35 -20 15, *t* = 5.96). **(b)** Changes in source activity (theta) observed at the left middle frontal gyrus (BA 46, peak MNI_x,y,z_= -45 35 20, *t* = 5.47). Slice views of source locations from left to right are axial, coronal, and sagittal images; viewed from top, back, and right. In all of the images, the left side of the brain is shown on the left. BA, Brodmann area

**Fig. 5:**
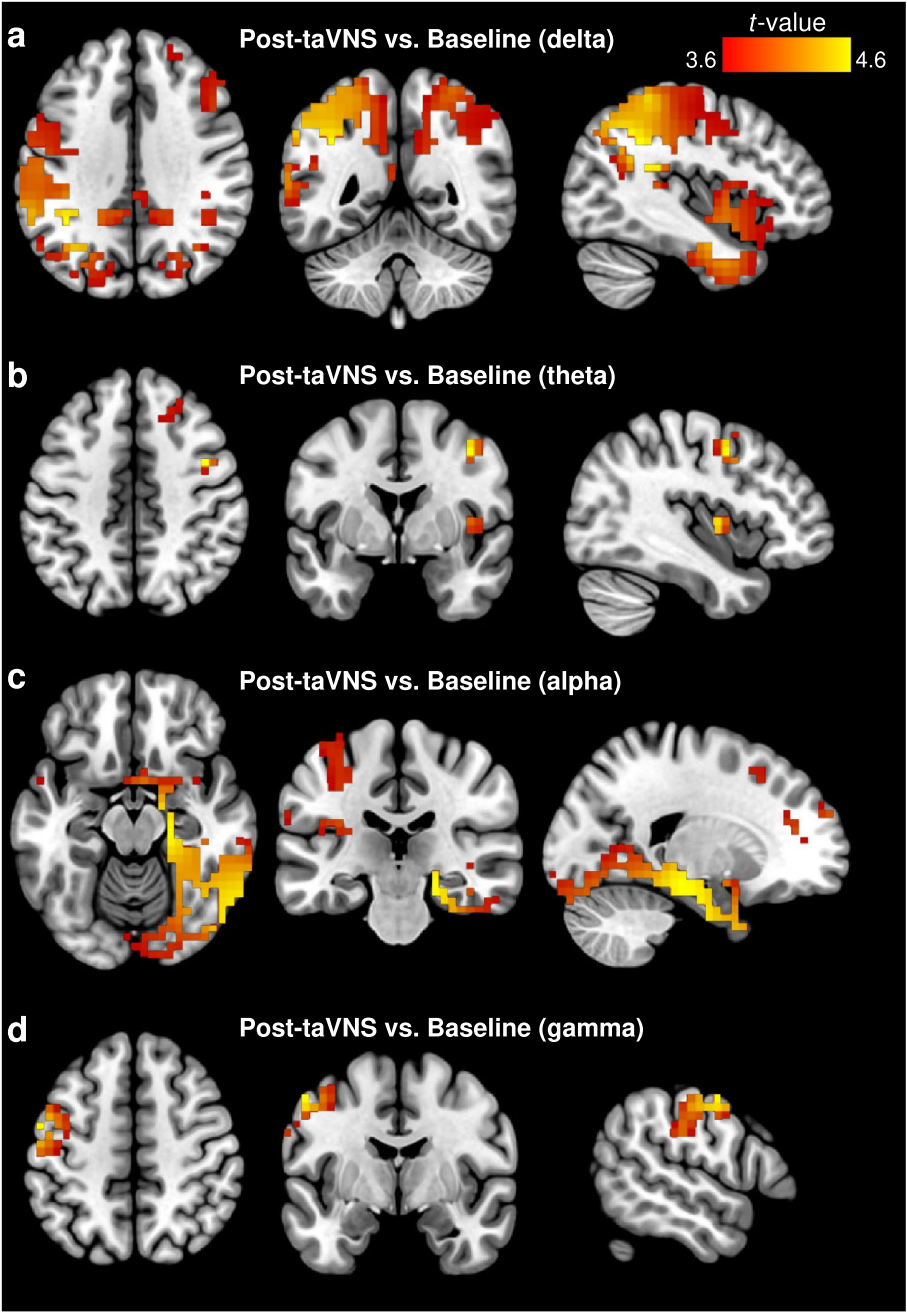
eLORETA of active Post-taVNS versus Baseline contrast. **(a)** Following taVNS, increased estimated source activity was observed at the supramarginal gyrus (BA 40, peak MNI_x,y,z_= -40 -50 35, *t* = 4.53) for delta, **(b)** the middle frontal gyrus (BA 6, peak MNI_x,y,z_= 40 0 45, *t* = 3.84) for theta, **(c)** the parahippocampal gyrus (BA 35, peak MNI_x,y,z_= 20 -25 -15, *t* = 4.62) for alpha, **(d)** and the precentral gyrus (BA 6, peak MNI_x,y,z_= -55 -5 50, *t* = 3.98) for gamma. Slice views of source locations from left to right are axial, coronal, and sagittal images; viewed from top, back, and right. In all of the images, the left side of the brain is shown on the left. taVNS, transcutaneous auricular vagus nerve stimulation; BA, Brodmann area

**Fig. 6:**
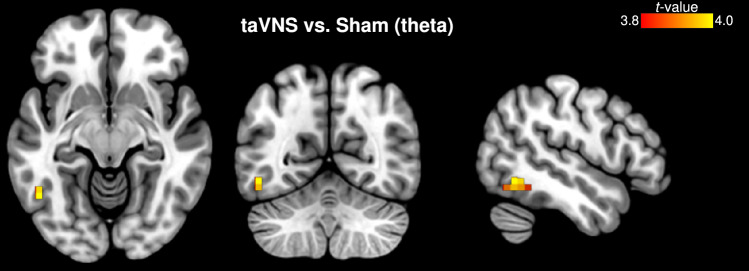
eLORETA of active taVNS versus Sham contrast. Differential source activity of theta oscillation was observed at the left middle occipital gyrus (BA 19, peak MNI_x,y,z_= -50 -60 -10, *t* = 3.97). Slice views of source locations from left to right are axial, coronal, and sagittal images; viewed from top, back, and right. In all of the images, the left side of the brain is shown on the left. taVNS, transcutaneous auricular vagus nerve stimulation; BA, Brodmann area

**Table 2:** Summary of brain regions responding to active taVNS and sham as well as following taVNS and sham electrical stimulation

			Location (MNI)			
Brain region	Brodmann area	Frequency	*x*	*y*	*z*	*t*-value
During-sham vs. Pre-sham						
R Insula, Sub-lobar	13	delta	35	$-20$	20	7.83
During-taVNS vs. Pre-taVNS						
R Insula, Sub-lobar	13	delta	35	$-20$	20	5.96
L Middle Frontal Gyrus, Frontal Lobe	46	theta	$-45$	35	20	5.47
taVNS (During-Pre) vs. sham (During-Pre)						
L Middle Occipital Gyrus, Occipital Lobe	19	theta	$-50$	$-60$	$-10$	3.97
Post-sham vs. Pre-sham						
R Middle Frontal Gyrus, Frontal Lobe	9	alpha	45	30	40	5.70
Post-taVNS vs. Pre-taVNS						
L Supramarginal Gyrus, Parietal Lobe	40	delta	$-40$	$-50$	35	4.53
R Middle Frontal Gyrus, Frontal Lobe	6	theta	40	0	45	3.84
R Parahippocampal Gyrus, Limbic Lobe	35	alpha	20	$-25$	$-15$	4.62
L Precentral Gyrus, Frontal Lobe	6	gamma	$-55$	$-5$	50	3.98

**Fig. 7:**
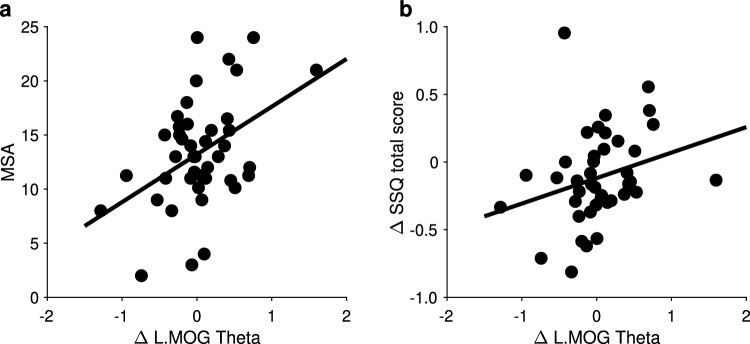
**(a)** Scatter plots show that the change in activation of the left middle occipital gyrus (L.MOG) between sham and taVNS stimulation was positively associated with MSA responses (Pearson *r* $= 0.43$), **(b)** and correlated with the change in SSQ total scores (Spearman $\rho$ $= 0.35$). MSSQ, motion sickness susceptibility questionnaire; MSA, below 12 years of age MSSQ scores; SSQ, simulator sickness questionnaire

Estimated source activity using eLORETA showed increased activation at the insula (BA 13, sub-lobar, MNI_x,y,z_= 35 -20 20, *t* = 7.83) when comparing sham to baseline at the delta band (Fig. 3a; Table 2). Of note, all brain images are presented in neurological convention; that is, the left side of the brain is shown on the left. Following sham stimulation, the middle frontal gyrus (BA 9, frontal lobe, MNI_x,y,z_= 45 30 40, *t* = 5.70) was more activated than at baseline at the EEG alpha band (Fig. 3b; Table 2).

During taVNS, the insula (BA 13, sub-lobar, MNI_x,y,z_= 35 -20 15, *t* = 5.96) and middle frontal gyrus (BA 46, frontal lobe, MNI_x,y,z_= -45 35 20, *t* = 5.47) showed a prominent increase in estimated source activity compared to baseline – at the delta and theta bands, respectively (Fig. 4; Table 2). Post taVNS administration, the supramarginal gyrus (BA 40, parietal lobe, MNI_x,y,z_= -40 -50 35, *t* = 4.53), middle frontal gyrus (BA 6, frontal lobe, MNI_x,y,z_= 40 0 45, *t* = 3.84), parahippocampal gyrus (BA 35, limbic lobe, MNI_x,y,z_= 20 -25 -15, *t* = 4.62), and precentral gyrus (BA 6, frontal lobe, MNI_x,y,z_= -55 -5 50, *t* = 3.98) all showed pronounced brain activation than at baseline, at the delta, theta, alpha and gamma bands, respectively (Fig. 5; Table 2).

The contrast (During-Pre)_taVNS_ vs. (During-Pre)_sham_ showed markedly increased theta brain activity in the left middle occipital gyrus (BA 19, occipital lobe, MNI_x,y,z_= -50 -60 -10, *t* = 3.97) (Fig. 6; Table 2); no significant effect of order was observed ($p = 0.6232$, unpaired-sample *t*-test). The observed taVNS-induced contribution of BA 19 theta effect correlated with reductions in motion sickness symptoms as evaluated by SSQ total scores (Spearman $\rho$ $= 0.35,\ p = 0.0229$; Fig. 7b). Additionally, this BA 19 activation was associated with MSA responses (Pearson *r* $= 0.43,\ p = 0.0041$; Fig. 7a). The (Post-Pre)_taVNS_ vs. (Post-Pre)_sham_ contrast did not reveal any significantly increased or decreased brain activation in the functional regions that were activated.

## Discussion

We conducted a crossover randomized controlled study to compare the acute effects of active taVNS (tragus stimulation) and sham (earlobe stimulation) administration on brain neural activation; simultaneous to motion-induced nausea provoked via a nauseogenic visual stimulus. To examine neural activation, we estimated electrical neuronal generators at the delta, theta, alpha, beta, and gamma EEG frequency bands using eLORETA – a technique that computes images of electric neuronal activity from EEG recordings. We show that when taVNS is administered during nauseogenic visual stimulation, participants exhibit significantly different functional brain activation in comparison to the sham condition. Moreover, we find that measures of the SSQ total, and SSQ nausea subscale were markedly lower when participants received taVNS compared to sham stimulation.

In both sham and taVNS conditions, we observed functional brain activation in the right insula (BA 13) during electrical stimulation. This heightened BA 13 activity was revealed as a strong contributor for delta oscillations, suggesting that the insula may play a shared or overlapping role under both active taVNS and sham conditions. While malaise development may be a possible explanation for insula activation in both these electrical stimulation conditions; given that the insula has been described among brain regions that engage in motion sickness generation (Napadow et al. 2013). During taVNS condition, insula activation may specifically serve as a response marker for taVNS-induced effects. Indeed, a meta-analysis of neuroimaging studies demonstrated that, when compared to null stimulation (i.e., no stimulation), transcutaneous vagus nerve stimulation significantly augmented activity in the insula (Rajiah et al. 2022).

Accompanying insula activation, increased neuronal response at the middle frontal gyrus (MFG; BA 46) was shown as a generator for theta oscillations during taVNS administration. The MFG resides in the frontal lobe; in fact, it is part of the dorsolateral prefrontal cortex (dlPFC) – a brain area with an essential role in higher-order cognitive control and function (Miller and Cohen 2001). Furthermore, the MFG has been associated with resolution of attentional-perceptual conflicts (Adelhöfer et al. 2019; Leroy and Cheron 2020). Whereas the insula has long been known to monitor internal body states (Damasio et al. 2000), and has been implicated in higher-order brain systems – one of which is the salience network (SN) (Seeley et al. 2007; Menon and Uddin 2010). The SN is a neural system involved in the integration of internal and external sensory inputs that vie for our brain’s attention, and neural resources. Taken together, we speculate that taVNS may be promoting salience processing among multisensory and cognitive domains by way of interoceptive signaling (Paciorek and Skora 2020). That is, it could be via this interoceptive afference and awareness, triggered by electrical stimulation of the vagus nerve, that participants become resilient and are provided the processing capacity to tolerate or buffer against the effects of the nauseogenic visual stimulation. During sham however, participants may be attaching ‘fear’ to the nauseogenic stimuli, hence, perceiving visual stimulation with translating stripes as a threat; similar to viewing of nausea-inducing motion video (Farmer et al. 2015). It should be noted that in the present study we also observed increased neuronal response in the anterior cingulate (see supplementary Fig. S3; BA 24, limbic lobe, MNI_x,y,z_= 5 35 10, *t* = 3.34) during sham condition and not during taVNS, although this activation was not significant; however, a finding plausible in light of evidence of disrupted anterior cingulate cortical response in motion-induced nausea (Napadow et al. 2013; Ruffle et al. 2019).

The most notable aspect of our findings was the peak neuronal response localized to the middle occipital gyrus (MOG; BA 19), when participants underwent active taVNS, in contrast to sham control stimulation (i.e., taVNS vs. sham contrast). This peak activation contributed to neuronal generation of theta oscillations (Fig. 6). Previous work in healthy participants undergoing fMRI scanning showed increased brain activity at the left occipital lobe when stimulation was applied at the surface of the neck, targeted at the cervical branch of the vagus nerve (Frangos and Komisaruk 2017). In another study, using high-resolution positron emission tomography (HR-PET) scanning, Wittbrodt et al. (2021) reported that BA 19 brain activity increased with application of tcVNS during exposure to traumatic stress scripts. In line with these reports, our findings indicate that similar regional brain activation can be observed via tragus stimulation triggering afferent signaling through the auricular branch of the vagus nerve. Moreover, the activation of BA 19 correlated with subjective measures of susceptibility to motion sickness (i.e., MSA scores). In addition to being correlated with reductions in the SSQ total score, suggesting that participants with greater BA 19 activity experienced significantly reduced symptoms of malaise. To interpret these results, we believe that the MOG (BA 19) may portray an essential role in describing effects that are a function of taVNS administration, at the same time as the presentation of the nauseogenic stimuli. The aversive experience of motion-induced nausea is complex, and characterized as a multidimensional perceptual state encompassing domains of cognition, emotion and interoception. Thus, it should be noted that we do not rule out the possibility that there may be other cerebral cortical regions that may have played a role in generation of effects affording participants to experience less malaise; as opposed to attributing these effects only to the MOG.

Anatomically, BA 19 resides in the extrastriate visual cortex – where the extrastriate body area (EBA) (Downing et al. 2001) can also be found. Astafiev et al. (2004) reported early fMRI evidence that the EBA responds strongly to body movements that are self-produced. We surmise here that the MOG neurons oscillate more strongly with taVNS, whereby participants generate neural representations to reestablish conflicting sensory signals from the viewpoint of an illusory body representation they perceive during moments where the nauseogenic stimulus overshadows taVNS signaling. That is, taVNS may be signaling higher-order brain structures to promote a sense of calm by stabilizing perception in situations of sensory conflict induced by translating visual stripes that provoke motion-related nausea. Wittbrodt et al. (2020) suggested a mechanism by which nVNS (i.e., tcVNS) activated the left fusiform gyrus (BA 20) – found in the temporal lobe – therefore implying that participants reconstructed their body form and environment during the traumatic event. In the same vein, we postulate taVNS influences the left MOG cortical activity to help manage motion-induced malaise. On the other hand, however, whether there is potential that modulation of neuronal activity in this cortical region may have therapeutic benefits for other nausea-related conditions, e.g., chemotherapy-induced nausea, remains unclear.

While increased neural activation following sham stimulation was much more widespread, the peak activation was located at the MFG (BA 9), serving as the generator of the alpha oscillations (Fig. 3). Like BA 46 (which had maximal activation during active taVNS; see above), BA 9 also resides in the dlPFC. Post-taVNS did not show a similar widespread increase of brain activity; rather, brain activation appeared more organized and localized (Fig. 5), suggesting that active taVNS and sham control influence neural activity differently following electrical stimulation for individuals who were simultaneously exposed to nausea-inducing stimuli. While this widespread activation following sham is ambiguous, we note our observation of dlPFC modulation during stimulation via taVNS, which we had ascribed to an indication that participants manage malaise through executive control by way of enhanced interoceptive processes; thus in this instance, it may imply that participants are recovering from the effects of nausea exposure. Because markedly increased sympathetic activation can be found after nauseogenic stimuli cessation (LaCount et al. 2011; Sclocco et al. 2016), another possible explanation may be that there are carry over effects of malaise. Indeed, this aforementioned dlPFC (MFG) activation has previously been implicated with increasing motion-induced nausea levels (Napadow et al. 2013); hence, we surmise here that these neural effects following sham may be indicative of response to motion-induced nausea – for instance, participants in recovery but still feeling nauseous – and not sham neural effects per se.

The greatest degree of taVNS influence on neural activation was observed during post-stimulation period; with taVNS contributing to EEG generators in all but one frequency band (i.e., EEG beta). We found peak neuronal activation at the supramarginal gyrus (SMG; BA 40), MFG (BA 6), parahippocampal gyrus (PHG; BA 35), and precentral gyrus (PCG; BA 6) (Fig. 5). Previously, SMG activation was reported in response to transcutaneous electrical stimulation (Frangos and Komisaruk 2017); however, that finding was observed during the stimulation period; additionally, electrical stimulation was achieved via the cervical branch of the vagus nerve. Our contrasting observation here may suggest vagal nerve branch specificity, that is, auricular versus cervical; or that vagal nerve-induced SMG response found here could be meaningful toward malaise-reduction effects; given the differences in protocol design. The SMG has a long identified role in vestibular processing; together with the angular gyrus, it forms part of the inferior parietal lobule. Klaus et al. (2020) demonstrated using vestibular stimulation, that the left inferior parietal lobule is involved in vestibular information processing, whereby the spatial processing of self-motion is linked with the spatial processing required to imagine self-motion. Because vestibular sensations are among some of the sensory information disrupted in the experience of motion sickness, by triggering the SMG, taVNS could be aiding maintain normal vestibular processing and function.

In early fMRI studies, the PHG – a limbic brain region implicated in emotion, and visuospatial processing, among other functions – was found to decrease in response to transcutaneous vagus nerve stimulation (Kraus et al. 2007, 2013). Interestingly, we found an increase in this brain region; this observation presumably links back to the notion of EBA, whereby in this instance participants become aware of their perceived body representation via visuospatial processing ability. A possible explanation for activation of these brain regions (i.e., SMG, MFG, PHG, PCG) may suggest a concerted multisensory integration to restore visuo-vestibular interaction profiles to a non-conflicting state. That active taVNS contributed to neural activation at four EEG frequency bands, whereas sham control influenced only a single EEG band, suggests that these neural changes may be specific only to stimulation via taVNS. Furthermore, it implies oscillations from these different bands may be working in concert to modulate neuronal activity across the frontal (MFG, PCG), parietal (SMG) and limbic (PHG) functional structures of the brain. Currently, it has been shown that motion-induced nausea augments functional connectivity between nausea-processing brain regions and those triggered by the nauseogenic visual stimulus (Toschi et al. 2017). Thus, for future research, it would be important to explore how connectivity analysis performed on a motion sickness model similar to that herein could aid deeper understanding of taVNS application to motion sickness. Collectively, these findings suggest a possible indication of taVNS-induced delayed effects after cessation of both the nauseogenic visual stimulation and transcutaneous vagus nerve stimulation. That is, while alpha and gamma oscillations did not show differential effects during the stimulation period, they did so during post-stimulation (Fig. 5). Corroborating these findings, delayed effects of taVNS on brainstem neuronal responses of healthy participants have been previously demonstrated with acute stimulation of the left cymba conchae (Borgmann et al. 2021).

Our observation that taVNS had differential effects on delta and theta oscillations both during and post-stimulation periods (Figs. 4 and 5), suggests that taVNS effects are sustained beyond stimulation period; giving the impression that taVNS-induced effects appear to last over time. Consistent with this, prior work has shown that vagal induced activity via the auricular branch of the vagus nerve persists after cessation of the stimulation (Frangos et al. 2015); however, it is worth noting in particular that our findings were not source-specific, but rather oscillation-specific. A possible explanation for these different findings may be differential site-specific vagal responses (i.e., cymba conchae versus tragus). Interestingly, however, Dimitrov and Gatev (2015) observed sustained delta and theta oscillations – in line with our findings – even after 20 min following right ear cymba conchae stimulation. Altogether, these findings may have implications for the role of taVNS in altering neural activity of these slow oscillations of the brain (i.e., delta, theta), with potentially long-lived effects, and merit further study.

When comparing post-taVNS versus post-sham, we found no differences in neural activity changes, to our surprise. This could mean that, in the context of motion-induced nausea, administering taVNS in real-time may provide neuromodulatory effects that present with neural properties that promote attenuation of malaise symptoms. It also suggests that these effects occur predominantly when the device is active (i.e., during stimulation period). These findings further endorse the viability of managing malaise in a manner whereby taVNS induces demand-based, modulatory effects.

Existing anti-motion sickness drugs such as anticholinergics (e.g., scopolamine), amphetamines (e.g., dextroamphetamine) and serotonin (e.g., rizatriptan) are effective at preventing or treating motion sickness, and do so by influencing the cholinergic (Kohl and Homick 1983), dopaminergic (Schmäl 2013), and serotonergic (Furman et al. 2011) pathways, respectively. Previous research performed on animal models has indicated that stimulating the vagus nerve engages these aforementioned brain pathways, i.e., cholinergic (Hulsey et al. 2016), dopaminergic (Perez et al. 2014), and serotonergic (Hulsey et al. 2019). In light of these reports, our neuronal activation findings herein may be a result of taVNS-induced alterations on neural processes of these neuromodulatory systems. Moreover, it reveals just how multifaceted the effects of electrically stimulating the vagus nerve may be.

Our study has several limitations. Firstly, while the taVNS protocol employed here influenced neural activation, our stimulation parameters and/or intensities may not have been optimal for alleviation of motion-induced nausea; optimization of the electrical stimulation parameters and intensity levels needs to be explored to fully unlock the potential of taVNS. Furthermore, because of the large number of female participants recruited in this study, our findings may be gender skewed. Exploring with a more gender-balanced participant cohort will improve the generalization of findings. Another potential future study would be to explore how combined stimulation of tragus and cymba conchae influences the experience of motion sickness. Finally, testing bilateral stimulation to the vagus nerve (i.e., performing stimulation on both ears to target the left and right auricular branches of the vagus nerve) could shed light on whether the effects improve, especially in exposure to increasing nausea-related stimuli.

## Conclusion

To our knowledge, this study explores for the first time whether taVNS, non-invasive electric vagal nerve stimulation via the auricular branch of the vagus nerve, contributes distinct functional brain activation (estimated by eLORETA) during, and after, coalesced exposure to visually induced motion sickness. Overall, the study showed deferential brain activation by verum in comparison to sham taVNS; and demonstrated a marked reduction in malaise severity following taVNS administration. These findings have implications for potential non-pharmaceutical strategies toward managing motion sickness, as well as for understanding the cerebral cortical activation through which taVNS may impart its effects.

## Supplementary Information

Below is the link to the electronic supplementary material.

Supplementary file 1 (pdf 954 KB)

## Data Availability

The datasets generated and analysed during the current study and the analysis code are available from the corresponding author upon reasonable request.
