# Supplementary material for: Transcutaneous Auricular Vagus Nerve Stimulation for Visually Induced Motion Sickness: An eLORETA Study
Source: Brain Topogr. 2024 Nov 2;38(1):11. doi: 10.1007/s10548-024-01088-6 (PMC11531436; doi:10.1007/s10548-024-01088-6)
Supplement: Supplementary file 1 — (pdf 954 KB) [file 10548_2024_1088_MOESM1_ESM.pdf]

Supplementary Material for

Transcutaneous Auricular Vagus Nerve Stimulation for Visually Induced Motion Sickness: An eLORETA Study

Emmanuel Molefi, Ian McLoughlin, and Ramaswamy Palaniappan

Correspondence: em576@kent.ac.uk

This PDF file includes:

Figs. S1 to S3
Table S1

Table S1. Tests for normality using Shapiro-Wilk test, and subsequent statistical analysis.

Location	Measure	Shapiro-Wilk	Statistical Test
Results text	Electrical stimulation	$p = 0.0631$	paired-sample t-test ($p = 0.8476$)
Fig. 1a	MSA and MSB	$p = 0.0004$	Wilcoxon signed rank test ($p = 0.2394$)
Fig. 1b	MSA and MSB correlation	$p = 0.0004$	Spearman $\rho = 0.35$ ($p = 0.0251$)
Fig. 7a	MSA and Δ L.MOG Theta correlation	$p = 0.3696$	Pearson $r = 0.43$ ($p = 0.0041$)
Fig. 7b	Δ SSQ total score and Δ L.MOG Theta correlation	$p = 0.0109$	Spearman $\rho = 0.35$ ($p = 0.0229$)

a totalmodel =

8×8 table

	SumSq	DF	MeanSq	F	pValue	pValueGG	pValueHF	pValueLB
(Intercept)	18.241	1	18.241	166.61	4.9762e-16	4.9762e-16	4.9762e-16	4.9762e-16
Error	4.489	41	0.10949					
(Intercept):Stimulation	0.78682	1	0.78682	32.022	1.3213e-06	1.3213e-06	1.3213e-06	1.3213e-06
Error(Stimulation)	1.0074	41	0.024571					
(Intercept):Time	3.0524	1	3.0524	63.371	7.518e-10	7.518e-10	7.518e-10	7.518e-10
Error(Time)	1.9748	41	0.048167					
(Intercept):Stimulation:Time	0.13206	1	0.13206	4.5849	0.038247	0.038247	0.038247	0.038247
Error(Stimulation:Time)	1.1809	41	0.028802					

b totalmodel_posthoc =

4×8 table

Time	Stimulation_1	Stimulation_2	Difference	StdErr	pValue	Lower	Upper
1	1	2	0.080798	0.03707	0.035083	0.0059345	0.15566
1	2	1	−0.080798	0.03707	0.035083	−0.15566	−0.0059345
2	1	2	0.19294	0.034167	1.3733e−06	0.12394	0.26195
2	2	1	−0.19294	0.034167	1.3733e−06	−0.26195	−0.12394

Fig. S1. (a,b) A two-way repeated measures ANOVA model output with post-hoc analysis for simulator sickness questionnaire (SSQ) total scores; comparing sham and active transcutaneous auricular vagus nerve stimulation (taVNS).

a nauseamodel =

8×8 table

	SumSq	DF	MeanSq	F	pValue	pValueGG	pValueHF	pValueLB
(Intercept)	13.698	1	13.698	145.74	4.4202e-15	4.4202e-15	4.4202e-15	4.4202e-15
Error	3.8534	41	0.093985					
(Intercept):Stimulation	0.88346	1	0.88346	21.472	3.6123e-05	3.6123e-05	3.6123e-05	3.6123e-05
Error(Stimulation)	1.6869	41	0.041145					
(Intercept):Time	2.0973	1	2.0973	41.341	1.0591e-07	1.0591e-07	1.0591e-07	1.0591e-07
Error(Time)	2.08	41	0.050733					
(Intercept):Stimulation:Time	0.17247	1	0.17247	5.7848	0.020763	0.020763	0.020763	0.020763
Error(Stimulation:Time)	1.2224	41	0.029814					

b nauseamodel_posthoc =

4×8 table

Time	Stimulation_1	Stimulation_2	Difference	StdErr	pValue	Lower	Upper
1	1	2	0.080952	0.04093	0.054694	−0.0017068	0.16361
1	2	1	−0.080952	0.04093	0.054694	−0.16361	0.0017068
2	1	2	0.20911	0.041277	9.0631e-06	0.12575	0.29247
2	2	1	−0.20911	0.041277	9.0631e-06	−0.29247	−0.12575

Fig. S2. (a,b) A two-way repeated measures ANOVA model output with post-hoc analysis for simulator sickness questionnaire (SSQ) nausea factor scores; comparing sham and active transcutaneous auricular vagus nerve stimulation (taVNS).

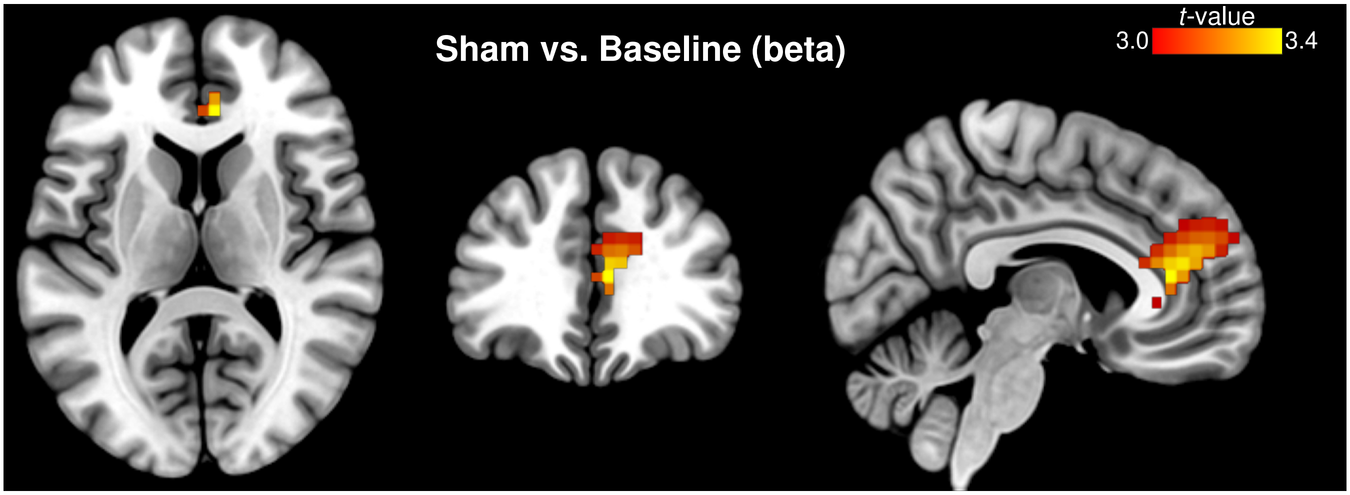

Fig. S3. Exact low-resolution brain electromagnetic tomography (eLORETA) of active Sham versus Baseline contrast. Differential source activity of beta oscillation was observed at the anterior cingulate (BA 24, limbic lobe, $MNI_{x,y,z}$= 5 35 10, t = 3.34). Slice views of source locations from left to right are axial, coronal, and sagittal images; viewed from top, back, and right. BA, Brodmann area.
